# Cost-Effectiveness of Lorlatinib as a First-Line Therapy for Untreated Advanced Anaplastic Lymphoma Kinase-Positive Non-Small Cell Lung Cancer

**DOI:** 10.3389/fonc.2021.684073

**Published:** 2021-05-28

**Authors:** SiNi Li, JianHe Li, LiuBao Peng, YaMin Li, XiaoMin Wan

**Affiliations:** ^1^ Clinical Nursing Teaching and Research Section, The Second Xiangya Hospital, Central South University, Changsha, China; ^2^ The Xiangya Nursing School, Central South University, Changsha, China; ^3^ Department of Pharmacy, The Second Xiangya Hospital, Central South University, Changsha, China

**Keywords:** cost-effectiveness (CE), non-small cell lung cancer, ALK, lorlatinib, crizotinib

## Abstract

**Introduction:**

Recently, a phase III CROWN trial compared the efficacy of two anaplastic lymphoma kinase (ALK) inhibitors and demonstrated that lorlatinib displayed clinical improvement over crizotinib for advanced non-small cell lung cancer (NSCLC) patients. Therefore, the aim of this study was to estimate the cost-effectiveness of lorlatinib as a first-line therapy for patients with advanced ALK-positive (+) NSCLC.

**Materials and Methods:**

A cost-effectiveness analysis was performed using a microsimulation model from the US payer perspective and a lifetime horizon (30 years) in patients with previous untreated advanced ALK+ NSCLC. Based on the CROWN trial, patient characteristics were obtained, and the transition probabilities were estimated. All direct costs were derived from official sources and published literature. The main outcomes of the model were total costs, incremental cost-effectiveness ratio (ICER), quality-adjusted life years (QALYs), and life years (LYs). One-way and probabilistic sensitivity analyses and multiple scenario analyses were conducted to test the robustness of the model outcomes.

**Results:**

In the base case analysis, in which 1 million patients were simulated, treatment with lorlatinib or crizotinib as the first-line treatment was related to a mean cost of $909,758 and $616,230 (incremental cost: $293,528) and a mean survival of 4.81 QALYs and 4.09 QALYs (incremental QALY: 0.72) per patient, respectively. The main drivers of cost effectiveness were drug price and subsequent cost. PAS indicated that lorlatinib has 90% cost-effectiveness when compared to crizotinib when the willingness-to-pay (WTP) threshold in increased to $448,000/QALY. Scenario analysis demonstrated that lorlatinib has 100% cost-effectiveness at a WTP threshold of 200,000/QALY compared to crizotinib treatment when the price of lorlatinib is decreased to 75% ($424.5) of its original price.

**Conclusions:**

In this study, lorlatinib was unlikely to be cost effective compared with crizotinib for patients with previously untreated advanced ALK+ NSCLC at a WTP threshold of 200,000/QALY.

## Highlights

1. This study reported that although lorlatinib significantly improved health outcomes, it still cannot be regarded as a cost-effective option compared with crizotinib for patients with untreated advanced ALK+ NSCLC from a US payer perspective.
2. When we adjusted the price of lorlatinib to $424.50, lorlatinib had 100% cost-effectiveness at a WTP threshold of 200,000/QALY compared with crizotinib treatment.
3. The implication of this study is not that crizotinib be used in place of lorlatinib or that lorlatinib should be withheld from patients. Rather, this study suggests that policymakers should control drug prices to within a reasonable range.

## Introduction

Lung cancer, a second most common cancer in the United States (US) among both men and women, has the greatest cancer-related mortality of all cancers in the US, accounting for almost 25% of all cancer deaths (1, 2). Non-small cell lung cancer (NSCLC) accounts for approximately 85% of lung cancer cases, and of these, approximately 2–7% are anaplastic lymphoma kinase (ALK), with the majority being of the nonsquamous subtype (3–5). The American Cancer Society reported that in 2020, 228,820 new lung cancer cases were diagnosed in the US, and 135,720 lung cancer deaths occurred (1). This formidable mortality is due mainly to a combination of the high incidence of lung cancer, and survival outcomes remain poor in patients with advanced lung cancer (i.e., stage III/IV): The 5-year relative survival for patients with distant metastasis is 5.8% (6, 7).

Although treatments for late-stage lung cancer are seldom curative, new therapies are urgently needed and have shown enormous potential for lung cancer patients in clinical practice (8, 9). Tyrosine kinase inhibitors (TKIs), the first targeted therapy for NSCLC, have demonstrated clinical improvements in both progression-free survival (PFS) and response levels and are thus recommended by clinical guidelines for patients with NSCLC (10–18). ALK rearrangement, a potential mechanism for targeted therapy was soon recommended for NSCLC treatment (19). Crizotinib, a first-generation targeted TKI for advanced ALK+ NSCLC, was approved by the US Food and Drug Administration (FDA) in 2011 and has been established as the current standard of care in the US (20, 21). Subsequently, although more potent ALK inhibitors (i.e., ensartinib, alectinib and brigatinib) have been developed and showed clinical improvement superior to that of crizotinib as a first-line therapy, crizotinib is still recommended as the standard of care for ALK+ patients in some countries worldwide because of pharma-economic evaluations (22–26). Lorlatinib, a third-generation ALK inhibitor, received approval from the US FDA in 2018 for the treatment of patients with advanced ALK+ NSCLC (27). Compared with crizotinib, lorlatinib is more potent in biochemical and cellular assays and has been identified as the agent with the broadest coverage of ALK-resistant mutations (28). Moreover, lorlatinib can achieve high exposure in the central nervous system because it can cross the blood–brain barrier (28).

Recently, the CROWN trial (NCT03052608), an international randomized phase III trial comparing lorlatinib with crizotinib in patients with previously untreated advanced ALK+ NSCLC, indicated that lorlatinib was associated with a significantly longer PFS, better quality of life (QoL), and a higher intracranial response rate (28). Seventy-eight percent (95% confidence interval [CI], 70–84) and 39% (95% CI, 30–48) of patients survived with progression-free disease at 12 months after lorlatinib and crizotinib treatment, respectively, and the hazard ratio was 0.28 (95% CI, 0.19–0.41, P <0.001) for disease progression or death (28). Previous studies have demonstrated that lorlatinib not only inhibits ALK more effectively than first- or second-generation inhibitors but also more potently treats central nervous system (CNS) metastases (29–32).

With targeted therapy becoming standard practice and the availability of an increasing number of novel therapeutic agents against ALK+ NSCLC, assessing the cost-effectiveness of new therapies has become instrumental in determining the implementation of these strategies. The aim of this study was to provide an economic evaluation of lorlatinib for advanced ALK+ NSCLC patients who had previously received no systemic treatment for metastatic disease to better understand its value from the US healthcare payer perspective.

## Materials and Methods

### Analytical Overview

**Figure 1:**
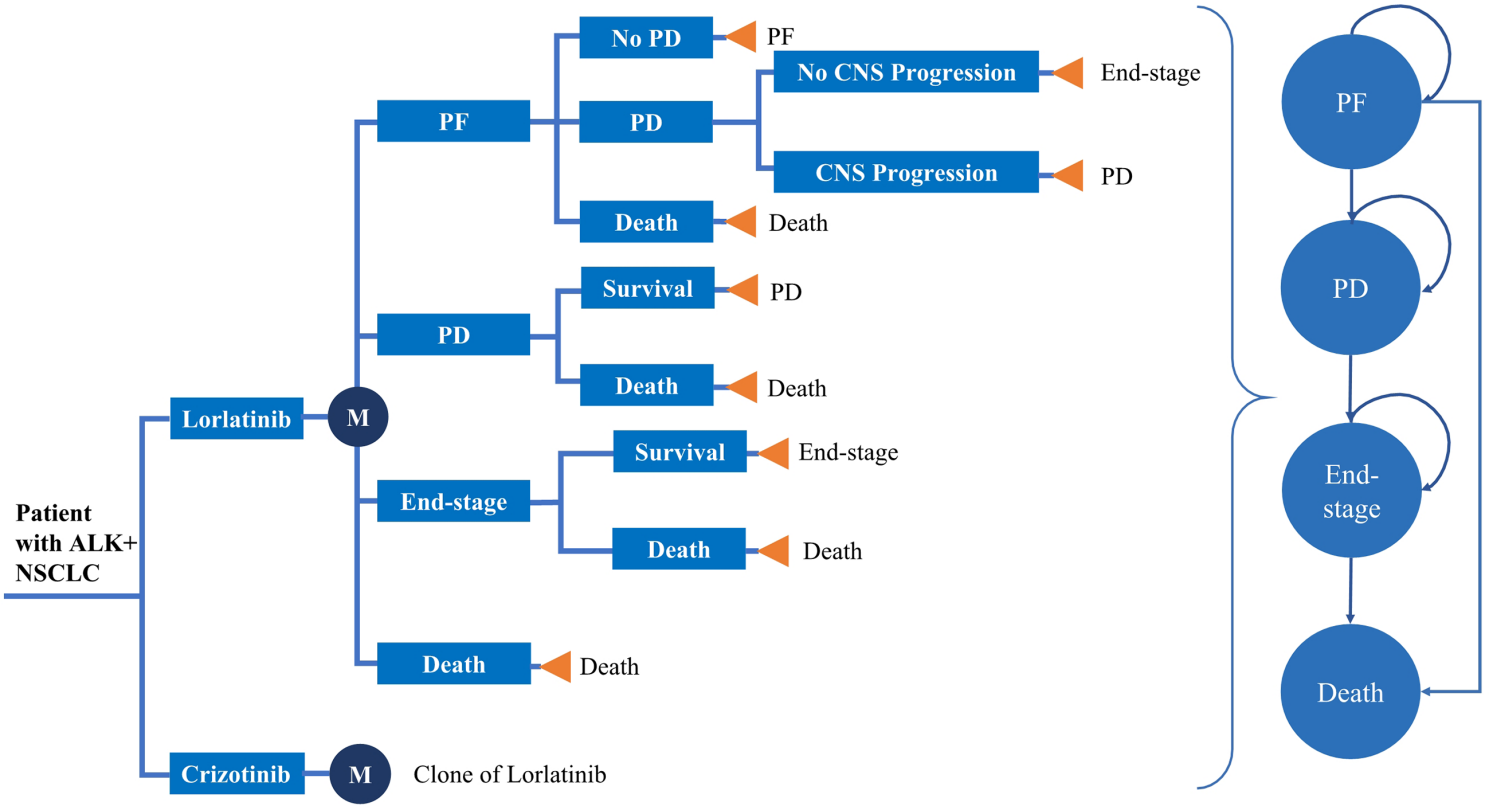
Model structure. *ALK, anaplastic lymphoma kinase; NSCLC, Non-small cell lung cancer; PF, progression-free; PD, progressive disease.

To reflect patient heterogeneity, a microsimulation model was developed to estimate the health and cost outcomes of patients with previously untreated advanced ALK+ NSCLC from the US healthcare payer perspective using TreeAge Pro software Version 2020. The model structure and input parameters were based on the results of the CROWN trial, previously published literature and publicly available US databases. The model included four mutually exclusive health states: Progression-free (PF), progression disease (PD), end-stage disease and death (**Figure 1**). All simulated patients entered the model in the PF health stage and could switch to PD, end-stage or death according to certain transition probabilities. Based on the CROWN trial, 2 treatment arms were included in the model, which simulated a 30-year horizon with a 28-day cycle length: First-line treatment with either oral lorlatinib (100 mg daily) or oral crizotinib (250 mg twice daily) until disease progression (See **Table S1** in the electronic **Supplementary Material** for details) (28). After disease progression, patients without CNS metastases in both the lorlatinib and crizotinib arms could receive subsequent therapy until death; otherwise, they could switch to end-stage and receive best supportive care (BSC). The main outcomes of this study were costs, quality-adjusted life years (QALYs), life years (LYs), and incremental cost-effectiveness ratios (ICERs). All cost and utility outcomes were discounted at 3% per year (33). A willingness-to-pay (WTP) threshold of $200,000/QALY was set when comparing the ICER between the two groups (34).

In the CROWN trial, a total of 296 patients were enrolled and randomly assigned to treatment with lorlatinib (n = 149) or crizotinib (n = 147). The patient characteristics are summarized in greater detail in **Supplementary Table S1**.

### Clinical Data Inputs

The Kaplan–Meier (KM) survival curves used to model overall survival (OS) and PFS were obtained from the CROWN trial using GetData Graph Digitizer version 2.26 to extract the data points. The probability of death in any state for lorlatinib and crizotinib use was estimated according to the OS curves of the CROWN trial. After we extracted the data points from the OS curves, the data of pseudoindividual patients were generated using an algorithm created by Hoyle et al. (35); then, five parametric survival models (exponential, Weibull, logistic, log-logistic, and lognormal) were used to fit the pseudoindividual patient. The results of the survival model fitting showed that an exponential distribution had the lowest Akaike information criterion and was regarded as the optimum model to fit the OS curves. The 2018 US life table was also used in the model to estimate the background mortality rate (36).

The transition probability of mortality between time t − *u* and t for the two strategies was calculated by using formula (1) below:

(1) $$Tp\left(t_{u}\right)=1-S\text{ (}t\text{)/}S\;(t-u)$$

while S(t) = exp(−λt) (λ > 0).

We used the same method to estimate the progression risk and probability of CNS metastases for lorlatinib and crizotinib based on the PFS curves from the CROWN trial. Exponential and Weibull distributions were considered the preferred models to fit the PFS curves for lorlatinib and crizotinib and were used to extrapolate progression rates.

### Cost and Utility Input

In this study, we assessed the aforementioned two treatments from the US healthcare payer perspective and thus only considered the following direct costs associated with cancer therapy: Drug acquisition, laboratory tests (37), monitoring for progression-disease (CT) (37), adverse events (AEs) management, BSC with or without CNS metastases, and subsequent therapy costs (38). All the costs were obtained from relevant US sources and corrected for inflation to reflect 2020 US dollars (39) (**Table 1**). The unit costs of the drugs were derived from First Data Bank, and the treatment costs per cycle were estimated using the unit cost and dosing schedules of the drugs on the basis of the average wholesale price minus 16% (**Tables 1**, **2**) (40, 41). The AEs included in the model were those with a severity of grade 3/4 and a frequency ≥5% or a difference of more than 2% between two treatment strategies in CROWN trial. We included hypercholesterolemia, hypertriglyceridemia, edema, and hypertension in the model and obtained the costs of AEs from previous studies (43–48). The CROWN trial reported the corresponding percentage of the population that received subsequent treatment, but it did not provide a specific protocol for subsequent treatment. Therefore, we used data from Deirdre F. Sheehan et al., whose study estimated the total cost of the following phase of care for lung cancer patients based on the Surveillance, Epidemiology, and End Results (SEER)-Medicare database to calculate the subsequent treatment cost in our study. Finally, based on the proportion of patients who received subsequent treatment in the lorlatinib group (69.1%) and the crizotinib group (24.5%) in the CROWN trial, we estimated that the subsequent costs of lorlatinib and crizotinib were $4,641 and $4,681 per cycle, respectively.

**Table 1:** Model parameters: baseline values, ranges, and distributions for sensitivity analysis.

Variable	Range				
	Baseline value	Minimum	Maximum	Distribution	Reference
Lorlatinib: Survival model					
OS	λ = −0.01968357 γ = −1.610781			Lognormal	Estimated (28)
PFS	λ = 0.008886453			Exponential	Estimated (28)
PFS of no CNS Progression	λ = 0.002070717			Exponential	Estimated (28)
Crizotinib: Survival model					
OS	λ = −0.110644 γ = 9.463829			Lognormal	Estimated (28)
PFS	λ = −0.01267741 γ = −2.117526			Lognormal	Estimated (28)
PFS of no CNS Progression	λ = −0.05653277 γ = −6.477169			Lognormal	Estimated (28)
Drug costs, per unit, (AWP-16%) $					
Lorlatinib, PO (100 mg)	566	453	680	Gamma	(40, 41)
Crizotinib, PO (250 mg)	257	206	308	Gamma	(40, 41)
Fenofibrate, PO (145 mg)	1.39	1.12	1.67	Gamma	(40, 41)
Lovastatin, PO (20 mg)	0.24	0.19	0.29	Gamma	(40, 41)
Support care costs, per week, $					
CNS metastases	3,538	2,830.4	4,245.6	Normal	Adjusted (42)
No CNS metastases	824.7	659.76	989.64	Normal	Adjusted (42)
Quality-of-life (utility)					
Progression free	0.81	0.79	0.84	Beta	(42)
Progression, second-line treated	0.72	0.70	0.75	Beta	(42)
Progression, best support care for CNS metastases	0.47	0.38	0.57	Beta	(42)
Lorlatinib: Incidence of AEs (%)					
Hypercholesterolemia	15	12	18	Beta	(28)
Hypertriglyceridemia	20	16	24	Beta	(28)
Edema	4	3.2	4.8	Beta	(28)
Hypertension	10	8	12	Beta	(28)
Crizotinib: Incidence of AEs (%)					
Hypercholesterolemia	0			Beta	(28)
Hypertriglyceridemia	0			Beta	(28)
Edema	1	0.8	1.2	Beta	(28)
Hypertension	0			Beta	(28)
AEs cost, $					
Hypercholesterolemia	8.12	6.496	9.744	Gamma	(40, 43, 44)
Hypertriglyceridemia	46.48	43.23	49.73	Gamma	(40, 45, 46)
Edema	2,623.65	2,098.92	3,148.38	Gamma	Adjusted (47)
Hypertension	9,410	7,528	11,292	Gamma	(48)
Discount rate	3	0	5	Uniform	
Subsequent therapy costs, $					
Lorlatinib	4,641	3,712.8	5,569.2	Gamma	Adjusted (28, 38)
Crizotinib	4,681	3,744.8	5,617.2	Gamma	Adjusted (28, 38)
CT per cycle	158	126.4	189.6	Gamma	Adjusted (37)
Laboratory	215	172	258	Gamma	Adjusted (37)

OS, Overall survival; PFS, Progression-free survival; CNS, Central nervous system; AWP, Average wholesale price; AEs, Adverse events.

**Table 2:** Summary base case results.

Results	Lorlatinib	Crizotinib	ICER
Total cost of regimen, $	909,758	616,230	
Life-years	6.25	5.45	
QALYs	4.81	4.09	
Per LY			368,211
Per QALY			409,667

ICER, incremental cost-effectiveness ratio; LY, life year; QALYs, quality-adjusted life years.

Utility values are often used to reflect a patient’s preference for living in a particular health state, with zero representing the worst health and one representing the best health (37). The CROWN trial did not report QoL results or outcomes. Therefore, we used utilities of 0.81 for patients in the PF phase and 0.72 for the PD phase, obtained from a previously published cost-effectiveness analysis with patient and disease characteristics similar to those of the CROWN trial (49). Patients who experience CNS metastases will ultimately switch to end-stage and receive BSC, so we used a utility of 0.47 for patients at that phase based on previous research conducted by Carlson (49) (**Table 1**).

### Analysis

To determine the key drivers of the model and to evaluate the robustness of the model, univariate deterministic sensitivity analysis (DSA), including 22 variables (costs, utilities, and risk of AEs) from the fitted extrapolative model, was performed. A probability sensitivity analysis (PSA) with 1,000 iterations of 10,000 patients was conducted to test the uncertainty of the model using second-order Monte Carlo simulation. In the sensitivity analysis, all parameters were assigned at a suitable distribution and were tested at the upper or lower limits of plausible ranges (**Table 1**) (42).

We also conducted multiple scenario analyses related to patient demographics, drug price, discount rate, utility value, and time horizon to assess how our assumptions affected the model outcomes. For example, in the scenario analyses, we not only considered the heterogeneity of NSCLC patients but also varied the drug costs of lorlatinib and crizotinib to evaluate the potential implications of drug tapering.

## Results

### Base Case Results

To deduce the effect of statistical fluctuations in the outcomes, 1 million patients were simulated for the two strategies, and the results are presented in **Table 2**. For lorlatinib, the mean cost and LYs were $909,758 and 6.25, respectively, while for crizotinib, the mean costs and LYs were $616,230 and 5.45, respectively. After adjustment for quality-adjusted life year (QALY), lorlatinib provided 4.81 QALYs, which was 0.72 QALYs more than for patients receiving crizotinib. The patients in the lorlatinib arm cost an additional $148,973, resulting in an ICER of $368,211/LYs or $409,667/QALYs compared with the crizotinib arm (**Table 2**).

### Sensitivity and Scenario Analysis

**Figure 2:**
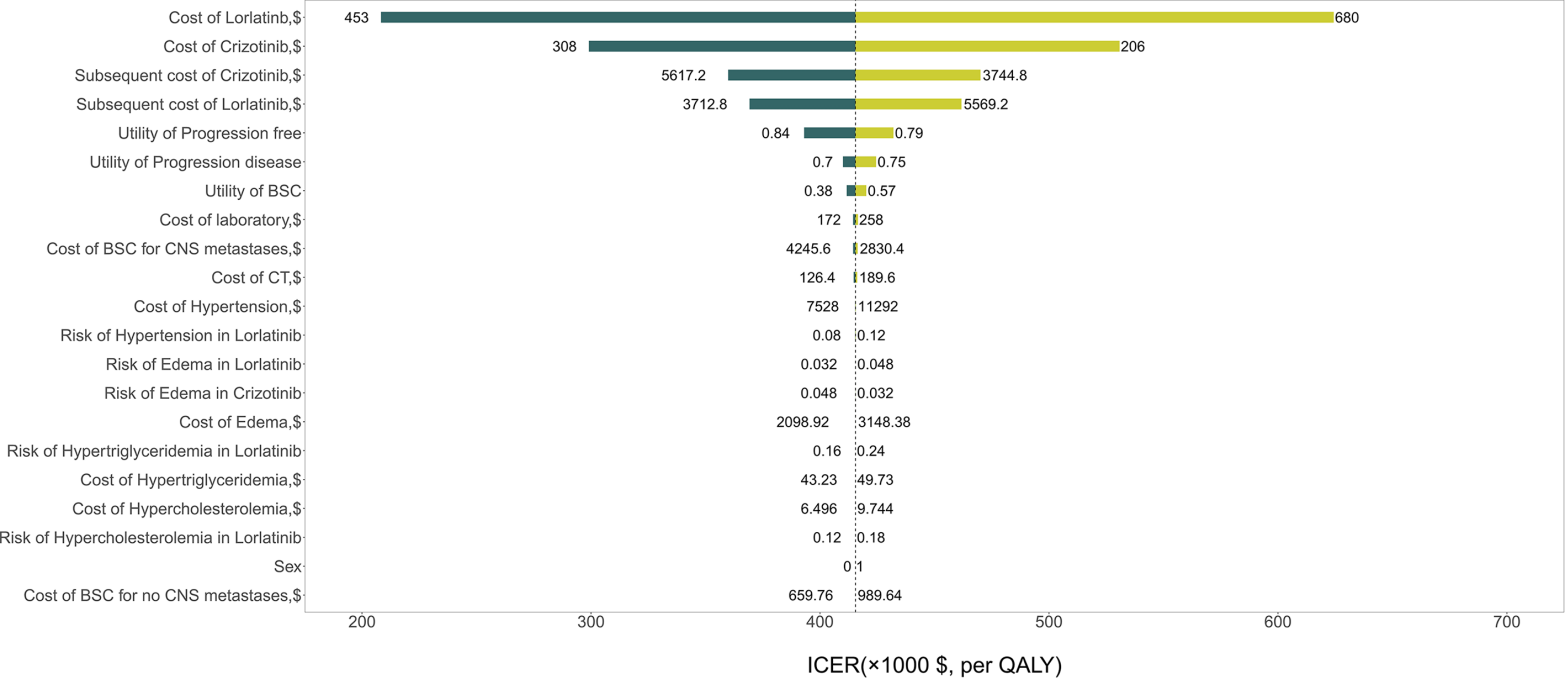
Tornado diagram for univariable sensitivity analysis. *ICER, Incremental cost-effectiveness ratio; BSC, Best supportive care; CNS, Central nervous system; AEs, Adverse events.

**Figure 3:**
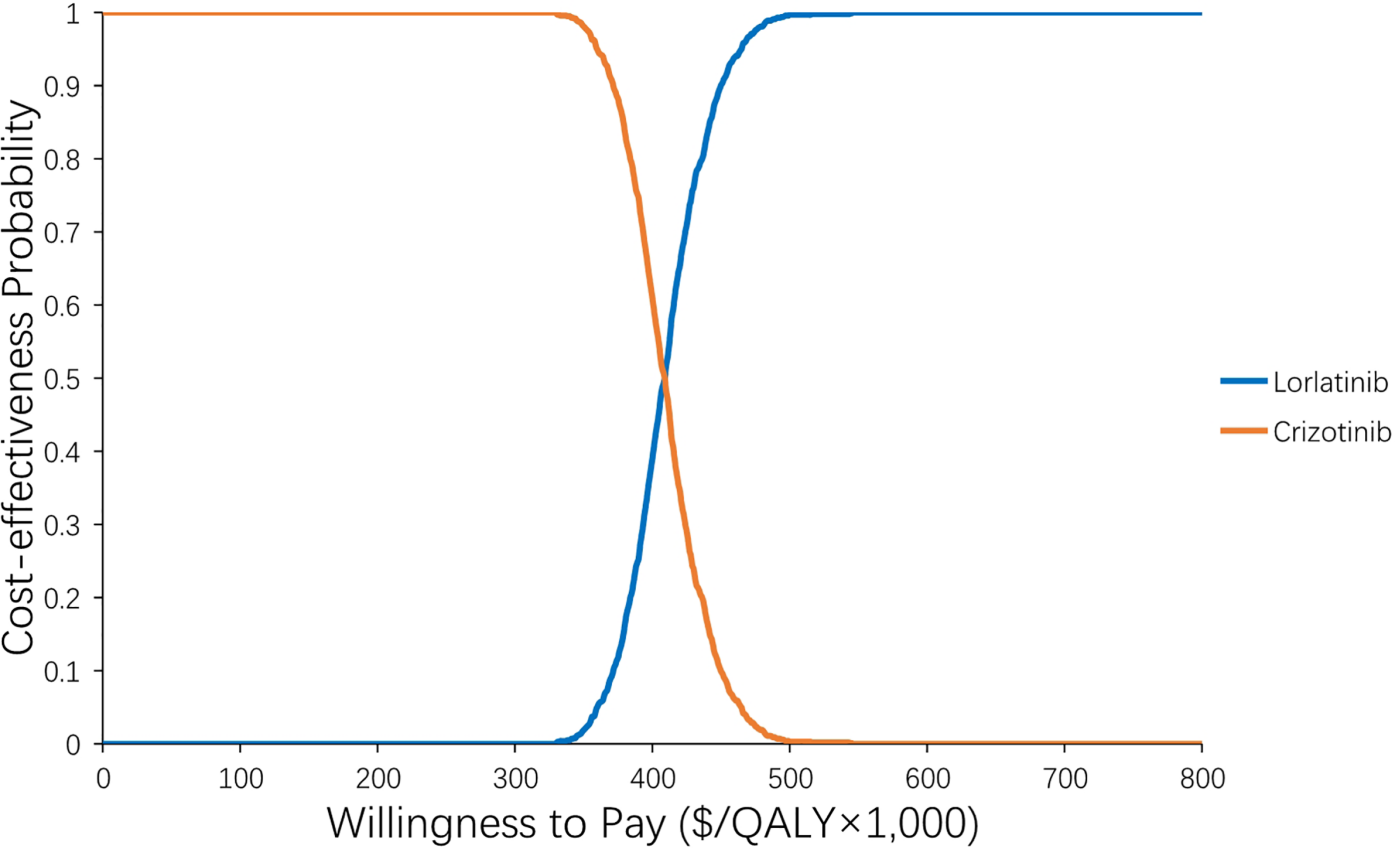
Acceptability curve of the probability sensitivity analysis. The probability sensitivity analysis of the base case.

The results of the one-way sensitivity analysis are presented in **Figure 2** and illustrate that the primary drivers of the model outcome were the drug prices of lorlatinib and crizotinib, the cost of subsequent treatment in the two strategies and the utility of PF. Other parameters, such as utility of PD, cost and risk of AEs, cost of BSC for CNS metastases, and sex, had moderate effects on the ICER. The PSA results in **Figure 3** show that without adjusting the drug price of lorlatinib, lorlatinib vs crizotinib had 90% cost-effectiveness only when the WTP threshold was increased to $448,000/QALY. Otherwise, it was impossible for lorlatinib to be cost-effective at the $200,000/QALY WTP threshold compared with crizotinib.

**Figure 4:**
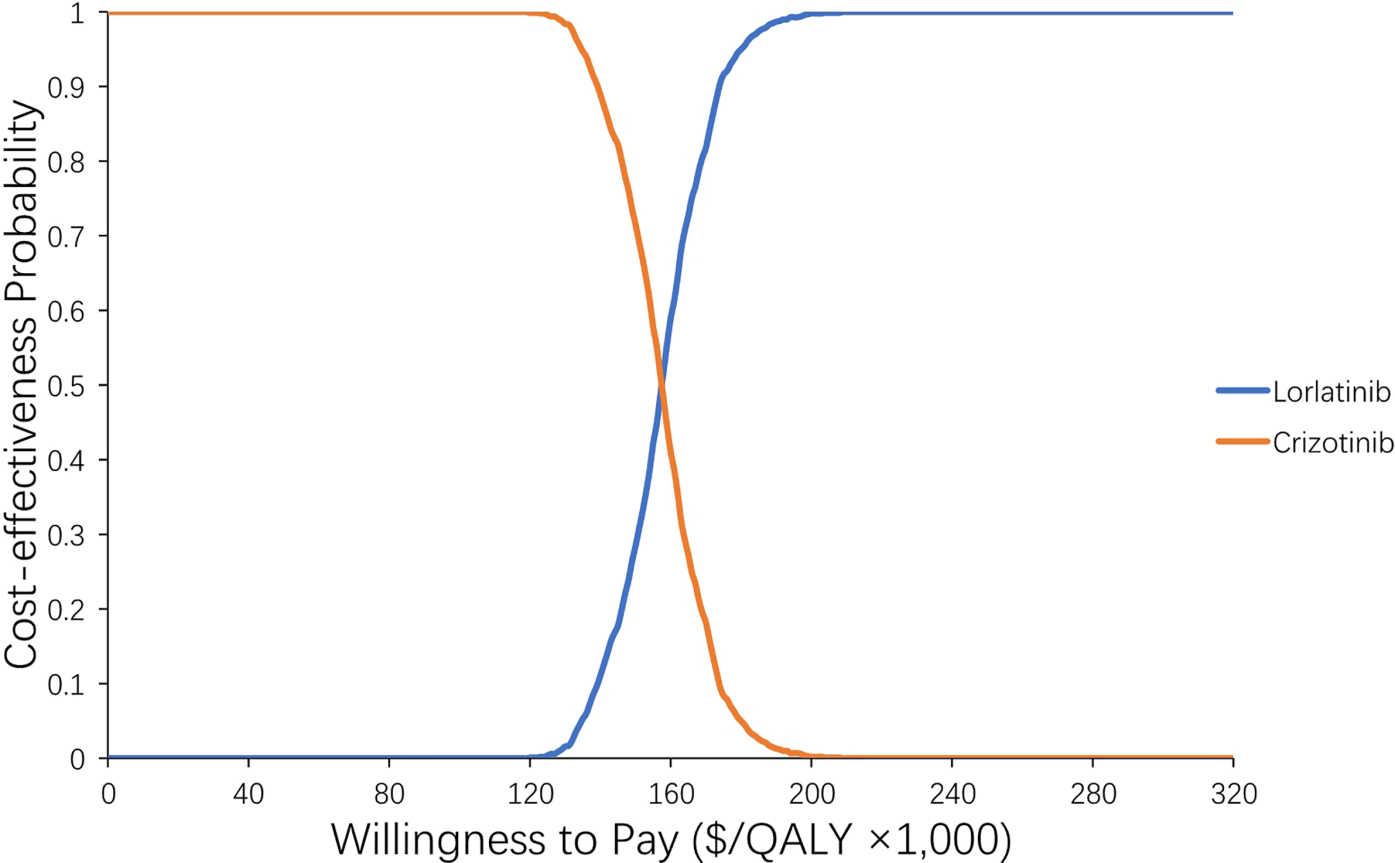
Acceptability curve of the probability sensitivity analysis. The probability sensitivity analysis of scenario 3-2 (adjusting the price of lorlatinib to its lower limit).

**Supplementary Table S4** shows the results of six scenario analyses. Notably, in scenario 3, when we adjusted the drug cost, the ICER for lorlatinib vs crizotinib treatment changed greatly. When the drug price of lorlatinib decreased to 75% ($424.5) of its original price, lorlatinib vs crizotinib treatment had 100% cost-effectiveness at a WTP threshold of 200,000/QALY, with a lower ICER of $161,154/QALY compared with the base case analysis (**Figure 4**). When we varied the drug price of lorlatinib to 50% ($283) and 25% ($141.5) of its original cost, the ICER for lorlatinib vs crizotinib therapy decreased to −$221,179/QALY and −$518,272/QALY, respectively. The negative ICER in the above cases confirms the dominance of the lorlatinib strategy, which accumulated higher QALYs at a lower cost over the model’s time horizon.

In scenario 6, the time horizon was changed to 5, 10 and 20 years to assess the impact of the OS and PFS extrapolations used in the model. Most of the costs (70%) occurred in the first 5 years of the time horizon; however, patient survival continued to increase after 5 years. Therefore, the longer the time horizon patients experienced, the greater their opportunity to accrue incremental benefit from disease progression and the lower the ICER obtained.

## Discussion

Over the past two decades, newly licensed anticancer drugs have been developed rapidly, which has been followed by an increase in the price of cancer drugs (50–52). Globally, the expenditure for anticancer drugs is approximately $100 billion annually, and the total expenditures for cancer have increased by a rate of 7.0% per year and are predicted to increase to $158 billion by 2025 (7, 50). The average treatment cost for a novel anticancer drug often exceeds $100,000 per year in the US (52). High drug prices not only increase patients’ out-of-pocket expenses, resulting in financial toxicity and low compliance, but also impose unsustainable cumulative price burdens for society (52). As a result, there is an urgent but challenging need to address extreme health care expenditures. To our knowledge, this is the first study worldwide to analyze the cost-effectiveness of a novel anticancer drug (lorlatinib) vs a standard-of-care drug (crizotinib) for the treatment of advanced ALK + NSCLC. The results revealed that compared with crizotinib, lorlatinib is unlikely to be cost effective in the current setting, although the acceptability of ICER values is subjective and depends on many other factors, such as social value and general budget (53). This lack of cost-effectiveness can be explained by the high cost of lorlatinib, since the scenario analysis and PSA results indicated that lorlatinib has 100% cost-effectiveness at a WTP threshold of 200,000/QALY vs crizotinib when the cost of lorlatinib is adjusted to the lower limit. Therefore, the implication of our study is not that lorlatinib should be withheld from patients with untreated ALK+ NSCLC; in particular, the advantages of lorlatinib treatment over crizotinib include slower progression of brain metastases for patients receiving long-term treatment (54). Rather, this study reveals the cost-effectiveness that would result from controlling the drug’s price to within a reasonable range. In the US, limited drug price transparency and the lack of unified government control over drug prices result in the highest drug costs in the world (55). Fortunately, the US government has proposed reducing the high drug costs paid by US patients by linking the drug prices paid by Medicare to those paid by health systems in other advanced countries (56). Once this plan is enacted or implemented, it might lower the price of lorlatinib and lead to more favorable economic outcomes.

The sensitivity analysis also illustrated that the subsequent cost greatly impacted the model outcome. Although the CROWN trial did not provide the specific treatment sequence after first-line treatment failure, we included possible clinical practices (BSC, surgery, chemotherapy and radiation) during the continuing treatment phase and calculated the subsequent treatment cost for patients with ALK+ NSCLC based on the previous study conducted by Deirdre. Therefore, we call for more RCTs to study the therapeutic sequence of ALK+ drugs in the future to help identify the best treatment sequence and offer the best QoL for patients with ALK+ NSCLC. At that time, we can further study the cost-effectiveness of lorlatinib as a first-line treatment or at any other point in the treatment sequence.

This research has certain limitations that merit mention. First, the main limitation of all cost-effectiveness studies is that they must adopt a particular set of circumstances and cannot widely and dynamically reflect the real-world clinical scenario (57). Cost-effectiveness studies will yield different outcomes when performed in different scenarios; for example, there is a large disparity between public and private health users in the US (58), and our study was conducted on the basis of the public health system. The study results cannot be generalized from one country to another due to the wide variation in healthcare systems among different countries. Second, the CROWN trial is the only randomized phase III trial that has directly compared lorlatinib and crizotinib for patients with advanced ALK+ NSCLC, and many input parameters (OS, PFS, AEs, etc.) in our model were obtained from this trial. Therefore, the external validity of our model largely depends on that trial, and any slight biases in that trial will have impacted our model outcome to some extent. Third, this study did not compare other potential treatment options due to a lack of head-to-head trials comparing multiple agents. Therefore, we call for more direct comparison trials of multiple potential treatment options in the future, and we will update our conclusion in the future if data are available. Fourth, owing to the lack of utility information in the CROWN trial, the utility values we used in our model were obtained from published cost-effectiveness studies that had the same patient characteristics as the CROWN trial. Although this may lead to some biases, we performed a series of sensitivity analyses that included wide variation in utility values. Finally, although it is usual in cost-effectiveness analyses to conduct an additional estimation to assess the financial consequences of adopting a new intervention (59, 60), we did not consider the budget impact that adding lorlatinib would have on society. However, the results of this evaluation might be a valuable reference for policymakers and physicians since it reflects the general clinical practice in managing advanced ALK+ NSCLC.

## Conclusion

From the US healthcare payer perspective, lorlatinib is determined not to be cost-effective when compared to crizotinib for NSCLC patients with previous untreated advanced ALK+ NSCLC at a willingness-to-pay threshold of $200,000 per QALY. However, when we decreased the drug price of lorlatinib to $424.50, the lorlatinib vs crizotinib strategy had 100% cost-effectiveness at a WTP threshold of 200,000/QALY. This implies that an appropriate drug price for lorlatinib should be taken into consideration when making policy decisions.

## Data Availability Statement

The original contributions presented in the study are included in the article/**Supplementary Material**. Further inquiries can be directed to the corresponding authors.

## Author Contributions

SL constructed the model, collected and analyzed the data, and drafted the manuscript. XW conceptualized the study and provided the model framework. YL contributed to the revision of the manuscript. LP and JL were the guarantors of the study and provided technical and material support. All authors contributed to the article and approved the submitted version.

## Funding

This study was funded by the National Natural Science Foundation of China (No: 71874209) and Hunan Provincial Natural Science Foundation of China (No. 2019JJ40411).

## Conflict of Interest

The authors declare that the research was conducted in the absence of any commercial or financial relationships that could be construed as a potential conflict of interest.
