# Supplementary material for: Cost-Effectiveness of Lorlatinib as a First-Line Therapy for Untreated Advanced Anaplastic Lymphoma Kinase-Positive Non-Small Cell Lung Cancer
Source: Front Oncol. 2021 May 28;11:684073. doi: 10.3389/fonc.2021.684073 (PMC8203315; doi:10.3389/fonc.2021.684073)
Supplement: Supplementary file 1 [file Table_1.docx]

Table S1. Patients Characteristics in CROWN trial

| Characteristic | Lorlatinib (N=149) | Crizotinib (N=147) |
| --- | --- | --- |
| Age, mean, yr | 59.1 (SD:13.1) | 55.6 (SD:13.5) |
| Sex, no. (%) |  |  |
| Female | 84 (56) | 91 (62) |
| Male | 65 (44) | 56 (38) |
| Race or ethnic group, no. (%) |  |  |
| White | 72 (48) | 72 (49) |
| Asian | 65 (44) | 65 (44) |
| Black | 0 | 1 (1) |
| Missing | 12 (8) | 9 (6) |
| ECOG performance-status score, no. (%) |  |  |
| 0 | 67 (45) | 57 (39) |
| 1 | 79 (53) | 81 (55) |
| 2 | 3 (2) | 9 (6) |
| Current stage of disease, no. (%) |  |  |
| IIIA | 1 (1) | 0 |
| IIIB | 12 (8) | 8 (5) |
| IV | 135 (91) | 139 (95) |
| Other | 1 (1) | 0 |

*ECOG: Eastern Cooperative Oncology Group

| Drug | Dose | Unit Cost (AWP-16%) | Treatment strategy | Total cost, 28days | Reference |
| --- | --- | --- | --- | --- | --- |
| Lorlatinib | 100mg | 566 | 100mg daily | 15848 | ^1,2^ |
| Crizotinib | 250mg | 257 | 250mg twice daily | 14392 | ^1,2^ |
| Fenofibrate (Treatment for hypertriglyceridemia) | 145mg | 1.39 | 145mg daily | 39.92 | ^1-4^ |
| Lovastatin (Treatment for Hypertriglyceridemia) | 20mg | 0.24 | 20mg daily | 6.72 | ^1,2,5,6^ |

Table S2. Dosage regimen.

*AWP: Average wholesale price.

Table S3. Significant predictors of cost during the continuing phase.

|  | N (%) | Total cost ($) | 95%CI |
| --- | --- | --- | --- |
| Stage III |  |  |  |
| BSC | 2255 (20.6) | 3817 | 3333-4301 |
| Surgery | 1395 (12.7) | 2761 | 2341-3181 |
| Radiation | 1813 (16.5) | 3909 | 3309-4508 |
| Chemotherapy | 1184 (10.8) | 6315 | 5525-7103 |
| Chemotherapy and radiation | 2738 (25.0) | 2759 | 2624-2895 |
| Stage IV |  |  |  |
| BSC | 893 (12.0) | 2983 | 2309-3657 |
| Radiation | 1091 (14.7) | 3084 | 2778-3389 |
| Chemotherapy | 1124 (15.1) | 5389 | 4645-6133 |
| Chemotherapy and radiation | 3512 (47.2) | 6181 | 5717-6644 |

*BSC: Best support care.

Table S4. Scenario analyses.

| Scenarios | Description | Cost | | QALY | | ICER |
| --- | --- | --- | --- | --- | --- | --- |
|  |  | Lorlatinib | Crizotinib | Lorlatinib | Crizotinib |  |
| 1-Baseline age | | | | | | |
| 1-1-Upper limit of baseline age  Base case = 59 (lorlatinib group)  Base case = 55 (crizotinib group) | Upper limit of age = 100 | 744362 | 425316 | 2.87 | 1.81 | 301235 |
| 1-2-Lower limit of baseline age  Base case = 59 (lorlatinib group)  Base case = 55 (crizotinib group) | Lower limit of age = 0 | 906005 | 589154 | 4.81 | 4.009 | 405788 |
| 2-Gender distribution | | | | | | |
| 2-1-All female simulation  Base case female proportion in lorlatinib group = 56%  Base case female proportion crizotinib group = 62% | Female proportion = 100% | 903173 | 612019 | 4.77 | 4.02 | 388205 |
| 2-2-All male simulation  Base case male proportion in lorlatinib group = 44%  Base case male proportion in crizotinib group = 38% | Male proportion = 100% | 899325 | 607084 | 4.76 | 4.02 | 394132 |
| 3-Costs | | | | | | |
| 3-1-Upper limit of Lorlatinib  Base case = 566 | Lorlatinib cost = 680 | 1058015 | 620344 | 4.80 | 4.10 | 528224 |
| 3-2-Lower limit of Lorlatinib  Base case = 566 | Lorlatinib cost = 453 | 759002 | 610043 | 4.79 | 4.06 | 205120 |
| 3-3-Upper limit of Crizotinib  Base case = 257 | Crizotinib cost = 308 | 910780 | 689666 | 4.83 | 4.09 | 297632 |
| 3-4-Lower limit of Crizotinib  Base case = 257 | Crizotinib cost = 206 | 907656 | 534483 | 4.78 | 4.07 | 526483 |
| 3-5-Cost of Lorlatinib is 25% lower than base case | Lorlatinib cost = 424.5 | 724088 | 605697 | 4.79 | 4.06 | 161154 |
| 3-6-Cost of Lorlatinib is 50% lower than base case | Lorlatinib cost = 283 | 551809 | 620517 | 4.85 | 4.14 | -97477 |
| 3-7-Cost of Lorlatinib is 75% lower than base case | Lorlatinib cost = 141.5 | 355982 | 609476 | 4.72 | 4.02 | -362853 |
| 4-Discount rate | | | | | | |
| 4-1- Higher discount rate  Base case discount rate = 3% | Discount rate = 5% | 829860 | 567867 | 4.31 | 3.70 | 427397 |
| 4-2- Lower discount rate  Base case discount rate = 3% | Discount rate = 0% | 1042131 | 687872 | 5.78 | 4.76 | 346402 |
| 5-Utility | | | | | | |
| 5-1- Upper limit of utility of PF  Base case value= 0.81 | Utility value = 0.84 | 912409 | 623232 | 4.96 | 4.22 | 391019 |
| 5-2- Lower limit of utility of PF  Base case value= 0.81 | Utility value = 0.79 | 921754 | 625786 | 4.77 | 4.10 | 440574 |
| 5-3- Upper limit of utility of PD (Treated)  Base case value= 0.72 | Utility value = 0.75 | 909094 | 612478 | 4.88 | 4.15 | 406323 |
| 5-4- Lower limit of utility of PD (Treated)  Base case value= 0.72 | Utility value = 0.70 | 907244 | 618242 | 4.76 | 4.06 | 412147 |
| 5-5- Upper limit of utility of PD (BSC)  Base case value= 0.47 | Utility value = 0.57 | 909665 | 616571 | 4.82 | 4.10 | 407075 |
| 5-6- Lower limit of utility of PD (BSC)  Base case value= 0.47 | Utility value = 0.38 | 909469 | 616674 | 4.81 | 4.10 | 412387 |
| 6-The time horizon | | | | | | |
| 6-1-Time horizon=5  Base case value=30 |  | 610488 | 457236 | 2.72 | 2.53 | 775826 |
| 6-2-Time horizon=10  Base case value=30 |  | 801426 | 559350 | 3.91 | 3.48 | 569626 |
| 6-3-Time horizon=20  Base case value=30 |  | 898720 | 607103 | 4.67 | 3.99 | 427057 |

1. Baseline age

Upper and lower baseline ages were explored in the 1-1 and 1-3 scenarios. Scenario 1-2 indicated that although lower age allowed patients could live with a longer life expectancy and they have more time and opportunity to accrue incremental benefit from disease progression, this also bring more medical expenditure for patients. So, the ICER for lorlatinib vs crizotinib therapy decreased slightly to $405788/QALY. Conversely, rising the baseline age to 100 meant less time to accrue and less cost to spend. The ICER for lorlatinib vs crizotinib was decreased to 301235/QALY.

2. Sex distribution

2-1 In the base case, the proportion of female was 56% in lorlatinib arm and 62% in crizotinib arm. We adjusted the proportion between male and female to explore the impact of sex distribution on outcome, increasing the proportion of females to 100%. This scenario leaded to a marginly lower ICER of $388205/QALY, compared to the base case.

2-2 When we assumed that 100% of patients were male, the ICER was slightly lower at $394132/QALY than in the base case.

3. Costs

We varied the costs of lorlatinib and crizotinib therapy from 3-1 to 3-7. As expected, a higher ($680) or lower ($435) lorlatinib drug cost led to a higher or lower ICER for lorlatinib vs crizotinib treatment of $528224/QALY or $205120/QALY. When the cost of lorlatinib was deduced to 25%, 50%, and 75% of the original price, the ICER for lorlatinib vs crizotinib therapy fell to $161154/QALY, $-97477/QALY, and -$362853/QALY, respectively.

4. Discount rate

In order to explore how discount rates affect the outcome of the model, a higher discount rate (5%) and no discount rate (0%) were used. The 4-1 scenario showed high discount rate caused a higher ICER of $427397/QALY; for a 0% discount rate scenario, a lower ICER of $346402/QALY for lorlatinib vs crizotinib therapy compared to the base-case analysis.

5. Utility

A higher baseline utility was assumed in this scenario to test the influence of this assumption on the model outcome. The results of 5-1, 5-3, and 5-5 showed that the higher baseline utility brings a higher QALY of 2 treatments, with a lower ICER of $391019/QALY, $406323/QALY, and $407075/QALY for lorlatinib vs crizotinib therapy compared to the base case.

When assuming a lower baseline utility for progression free, progression disease with second-line therapy treated, and progression disease with best support care for CNS, the ICER of lorlatinib vs crizotinib therapy increased to $440574/QALY, $412147/QALY, and $412387/QALY, respectively, compared with the baseline case.

6. Time horizon

We changed the time horizon in 6-1 to 6-3. The ICER ranged from $427057/QALY for 20 years to $775826/QALY for 5 years. when patients obtained more treatment time, more chance to accrue incremental benefit from disease progression and the ICER becomes smaller.

References:

1. First DataBank Inc. Analy$ource Online: the online resource for drug pricing and deal information. 2020. <https://www.analysource.com>.

2. Curtiss FR, Lettrich P, Fairman KA. What is the price benchmark to replace average wholesale price (AWP)? *Journal of managed care pharmacy : JMCP.* 2010;16(7):492-501.

3. Sidhu G, Tripp J. Fenofibrate. In: *StatPearls.* Treasure Island (FL)2020.

4. Yuan G, Al-Shali KZ, Hegele RA. Hypertriglyceridemia: its etiology, effects and treatment. *CMAJ.* 2007;176(8):1113-1120.

5. Duong H, Bajaj T. Lovastatin. In: *StatPearls.* Treasure Island (FL)2020.

6. Benner JS, Smith TW, Klingman D, et al. Cost-effectiveness of rosuvastatin compared with other statins from a managed care perspective. *Value in health : the journal of the International Society for Pharmacoeconomics and Outcomes Research.* 2005;8(6):618-628.
